# A systematic review of guidelines for managing rheumatoid arthritis

**DOI:** 10.1186/s41927-019-0090-7

**Published:** 2019-10-22

**Authors:** Aneela Mian, Fowzia Ibrahim, David L. Scott

**Affiliations:** Academic Rheumatology, Department of Inflammation Biology, School of Immunology And Microbial Sciences, King’s College London, Weston Education Centre, Denmark Hill, London, SE5 9RT UK

**Keywords:** Rheumatoid arthritis, Systematic review, Management guidelines

## Abstract

**Background:**

We systematically reviewed current guidelines for managing rheumatoid arthritis (RA) to evaluate their range and nature, assess variations in their recommendations and highlight divergence in their perspectives.

**Methods:**

We searched Medline and Embase databases using the terms ‘clinical practice guidelines’ and ‘rheumatoid arthritis’ from January 2000 to January 2017 together with publications of national and international bodies. We included guidelines providing recommendations on general RA management spanning a range of treatments and published in English. We undertook narrative assessments due to the heterogeneity of the guidelines.

**Results:**

We identified 529 articles; 22 met our inclusion criteria. They were primarily developed by rheumatologists with variable involvement of patient and other experts. Three dealt with early RA, one established RA and 18 all patients. Most guidelines recommend regular assessments based on the Outcome Measures in Rheumatology core dataset; 18 recommended the disease activity score for 28 joints. Twenty recommended targeting remission; 16 suggested low disease activity as alternative. All guidelines recommend treating active RA; 13 made recommendations for moderate disease. The 21 guidelines considering early RA all recommended starting disease modifying drugs (DMARDs) as soon as possible; methotrexate was recommended for most patients. Nineteen recommended combination DMARDs when patients failed to respond fully to monotherapy and biologics were not necessarily indicated. Twenty made recommendations about biologics invariably suggesting their use after failing conventional DMARDs, particularly methotrexate. Most did not make specific recommendations about using one class of biologics preferentially. Eight recommended tapering biologics when patients achieved sustained good responses.

**Conclusions:**

Five general principles transcend most guidelines: DMARDs should be started as soon as possible after the diagnosis; methotrexate is the best initial treatment; disease activity should be regularly monitored; give biologics to patients with persistently active disease who have already received methotrexate; remission or low disease activity are the preferred treatment target.

## Background

Guidelines for the management of rheumatoid arthritis (RA) produced by expert groups based on assessments of the research evidence have been produced for over 25 years [1–4]. They provide explicit recommendations to influence practice through a formal process of disseminating advice on effective management. Guidelines can help minimise unnecessary care. Many guidelines for managing RA have been published over recent years; many of them have been updated to take into account new treatments and novel research evidence about existing treatments.

The existence of multiple guidelines raises several questions. First, as they have all had access to the same research data, albeit at different time-points, are there recommendations similar or are there substantial differences between them? Second, why are there different guidelines dealing with the same issue – how best to treat RA? Thirdly, what is the impact of these guidelines on clinical practice? Finally, what guidelines will be needed in future years?

We have systematically reviewed current RA guidelines. Our overall aims were to evaluate the range and nature of guidelines currently available, to assess the variations in their recommendations about RA management, and highlight any divergence in their perspectives. The specific questions we considered were: (a) to examine their recommendations about composite assessments of disease activity; (b) to identify their management targets with drug therapy; (c) to define the categories of drug treatments considered. As a consequence of these assessments we sought to provide insights into the value and relevance of different guidelines.

## Methods

### Literature search

We searched Medline and Embase databases using the terms ‘clinical practice guidelines’ and ‘rheumatoid arthritis’. We also searched national bodies including the Scottish Intercollegiate Guidelines Network (SIGN) and the National Institute For Health and Care Excellence and national and international specialist societies including the British Society for Rheumatology, the American College of Rheumatology and the European League Against Rheumatism. Finally we searched lists of references from identified guidelines.

### Inclusion and exclusion criteria

Our inclusion criteria comprised: (a) publications that identified themselves as guidelines; (b) guidelines that provided recommendations on the general management of RA; (c) guidelines that included a range of different drug treatments; (d) guidelines published from January 2000 to January 2017; (e) guidelines published in English. Our exclusion criteria comprised: (a) guidelines and appraisals that dealt with specific areas of management, such as safety monitoring of drugs; (b) guidelines or appraisals of single drugs or technologies. When there were several versions of guidelines from the same organisation, only the latest guideline was included.

### Screening and data extraction

Two researchers (AM, DLS) independently assessed studies for eligibility and extracted data onto a predefined template. The data included: (a) year of publication; (b) format (who was involved); (c) quality method followed; (d) systematic review of evidence; (e) patient groups considered; (f) area of management included; (g) composite activity assessments; (h) prognostic assessments; (i) treatment targets; (j) and range of treatments considered. When there were differences between assessors, they reviewed the reports together and came to a joint conclusion.

### Assessment of quality methods

We sought evidence that individual guidelines had followed nationally or internationally accepted quality methods in their development; we did not assess their quality as part of this report. Firstly, we recorded who had been involved in developing the guideline, including the involvement of specialists, other experts and patients. Secondly we evaluated whether they had used recognised quality methods such as Agree and Agree II [5], Adapte [6], Grade [7], and National Institute for Health and Clinical Excellence (NICE) [8] methods. Thirdly we sought evidence whether they had used systematic reviews of published evidence to develop their recommendations. We did not specifically examine the quality of individual guidelines because we anticipated this would be highly variable because some guidelines were developed by large organisations such as the American College of Rheumatology whilst others were developed by smaller groups with far less resources making substantial variations in the quality of the guidelines inevitable.

### Methodological approaches

We followed the general PRISMA recommendations [9] and other approaches for systematic reviews [10], although none of these specifically deal with reviews of guidelines. We also followed methods recommended for reviews of systematic reviews [11] and approaches taken in previous systematic reviews of guidelines [12, 13]. As PRISMA does not specifically include systematic reviews of guidelines we did not pre-register our protocol; this was omitted in other systematic reviews of guidelines [12].

### Methods of analysis

The guidelines were very heterogeneous in terms of the areas covered, the approaches taken in their development and the presentation of their recommendations. Consequently we undertook narrative assessments of their recommendations. Initially we assessed the areas covered by the guidelines, whether they included statements of principles and needs, their intended audiences and their overall structure, including whether they dealt with specific questions or recommendations. We then focussed on three predefined areas related to our specific aims. These comprised; (a) recommendations about composite assessments of disease activity and other assessments; (b) management targets with drug therapy including the impact of prognostic assessments; (c) and the categories of drug treatments considered. We considered this approach would enable us to assess the variations in their recommendations about RA management and identify divergences in their perspectives. We did not set out to produce any single optimal set of recommendations for RA management from our analyses of these guidelines. We considered management from the perspective of conventional disease modifying anti-rheumatic drugs (DMARDs) like methotrexate, biologic DMARDs like tumour necrosis factor inhibitors, Janus Kinase (JAK) inhibitors and glucocorticoids (steroids).

## Results

### Guidelines identified

**Fig. 1:**
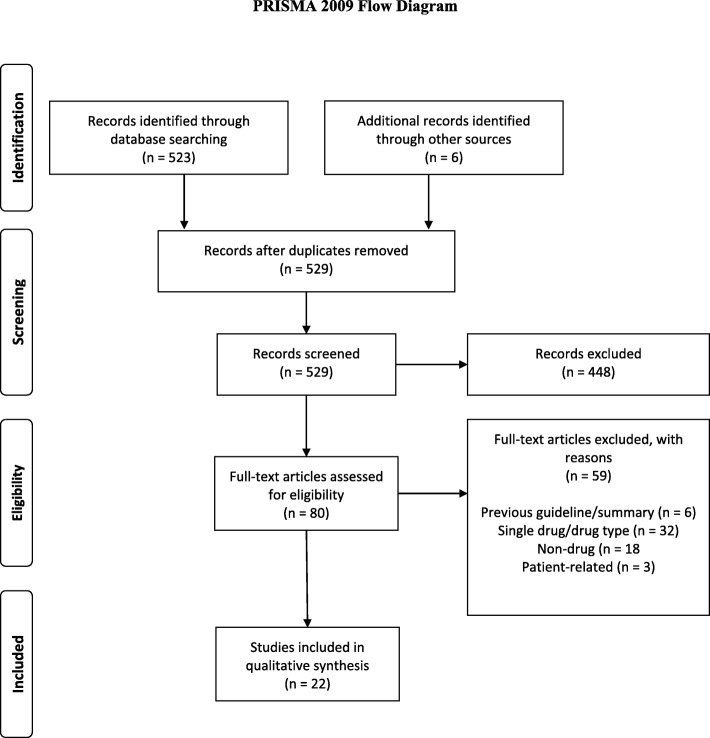
PRISMA 2009 Flow Diagram

We identified 529 potential guidelines articles: 80 were assessed in detail; 22 guidelines [14–35] selected because they met our inclusion criteria (Fig. 1). These included two European League Against Rheumatism (EULAR) guidelines, which provided general guidance and guidance of treat to target [22, 34], and four different guidelines from the United Kingdom [6, 7, 24, 25], which were produced by various groups at different times and worked from varying perspectives.

The 59 excluded guidelines articles included 5 superseded guidelines and one separately published summary article, 32 guidelines that dealt with single drugs or drug classes, 18 that dealt with non-drug treatments and 3 patient-related articles.

### Features of guidelines

These are summarised in Table 1. Groups of expert rheumatologists were reported as drawing up 21/22 guidelines; the only exception was the British Columbia guidelines, which did not specify who was involved in their construction [18]. There were variable levels of patient involvement; 12/22 guidelines specified there was patient involvement [14–16, 19–24, 31, 34]. There were also variable levels of contributions from other experts, such as nurses, other allied health professionals, experts in systematic reviews and a range of other areas; such experts were involved in 12/22 guidelines [14, 16, 19–23, 29–32, 35].

**Table 1:** Features of clinical guidelines included in review

Guideline	Year	Format			Quality Method	Systematic Review Of Evidence		Patients	Areas Covered		
		Specialists	Other Experts	Patients		In Guideline	Separate		Diagnosis	Drugs	MDT
1. American [14]	2015	Yes	Yes	Yes	Grade	Yes	–	All	–	Yes	–
2. APLAR [15]	2015	Yes	–	Yes	Agree II	Yes^a^	–	All	–	Yes	–
3. Australian [16]	2009	Yes	Yes	Yes	Agree	Yes^a^	–	< 2 years	Yes	Yes	Yes
4. Brazilian [17]	2012	Yes	–	–	–	Yes	–	All	–	Yes	Yes
5. British Columbia [18]	2012	Not Specified			–	–	–	All	Yes	Yes	Some
6. British Society For Rheumatology: Established [19]	2009	Yes	Yes	Yes	–	–	–	> 2 years	–	Yes	Yes
7. British Society For Rheumatology: Early [20]	2010	Yes	Yes	Yes	–	–	–	< 2 years	Yes	Yes	Yes
8. Canadian [21]	2011	Yes	Yes	Yes	Adapte	Yes^a^		All	–	Yes	–
9. EULAR [22]	2016	Yes	Yes	Yes	Agree II	–	Yes	All	–	Yes	–
10. French [23]	2014	Yes	Yes	Yes	–	–	–	All	Yes	Yes	Some
11. German [24]	2013	Yes	–	Yes	–	Yes		All		Yes	–
12. Hong Kong [25]	2010	Yes	Not Specified		–	–	–	All	Yes	Yes	–
13. Indian [26]	2008	Yes	Not Specified		–	–	–	All	Yes	Yes	Yes
14. Latin American [27]	2006	Yes	Not Specified		–	–	–	All	Yes	Yes	Yes
15. Mexican [28]	2014	Yes	Not Specified		Agree II	Yes^a^	–	All		Yes	
16. England [29]	2009	Yes	Yes	Yes	NICE	Yes	–	All	Yes	Yes	Yes
17. Scotland [30]	2011	Yes	Yes	–	Grade	Yes^b^		< 5 years	Yes	Yes	Yes
18. South African [31]	2013	Yes	Yes	Yes	–	–	–	All	Yes	Yes	Yes
19. Spanish [32]	2007	Yes	Yes	–	–	Yes		All	Yes	Yes	Yes
20. Swedish [33]	2011	Yes	Not Specified		–	–	–	All	–	Yes	–
21. Treat to Target [34]	2010	Yes	–	Yes	–		Yes	All	–	Yes	–
22. Turkish [35]	2011	Yes	Yes	–	–	Yes		All	–	Yes	Yes

*MDT* Multidisciplinary team

^a^Systematically reviewed other guidelines

^b^used existing published systematic reviews

The guidelines varied substantially in the ways they were constructed. Three guidelines [15, 22, 28] used Agree II methods and one guideline [16] the Agree method, two used Grade methods [14, 30], one guideline [29] used NICE methods and one guideline [21] the Adapte method. Although other guidelines did not use any formal guidelines methods, in many instances they were intended to amend existing international guidelines for local circumstances.

The approaches to assessing clinical research evidence supporting the guidelines also varied. The two EULAR guidelines [22, 34] commissioned detailed systematic reviews which were published separately [36, 37]. The American College of Rheumatology (ACR) guideline commissioned [14] detailed systematic reviews that were published as an appendix. The English (Royal College of Physicians) guideline [29] commissioned detailed systematic reviews for each question which were published within the guideline itself. Eight other guide guidelines included some systematic reviews [15–17, 21, 24, 28, 30, 32, 35, 38] within them, including systematically assessing other guidelines, and one other guideline formally used existing published systematic reviews to assess each question they considered [30].

Two guidelines dealt with early RA under 2 years duration [16, 20] and one under 5 years duration [30]; one guideline dealt with established RA over 2 years [19] duration; the other guidelines dealt with all RA patients.

### Areas covered

All the guidelines dealt with drug treatment, though they did not all cover the same aspects of drug therapy. Eleven guidelines also covered diagnosis [16, 18, 23, 25–27, 29–32] and 13 covered some or many non-drug treatments [16–20, 23, 27, 29–32, 35]. Those guidelines which considered non-drug treatments by multidisciplinary teams outlined a range of supportive treatment options. Some of these guidelines, such as the Spanish guidelines [32], provided extensive details about these non-drug treatments. Others, such as the Scottish guidelines [30], give more general recommendations.

### Statements of principles and needs

Guidelines often included a range of statements of general principles, the specific need for the guideline and the audience the guideline was intended to inform. These statements were so diverse that it is not possible to provide a succinct summary of them.

The EULAR guidelines [22] provided the most extensive global statements which were mainly related to ethical issues and philosophical principles such as the central role of patients, the role of specialist rheumatologists and the high costs of the disease burden in RA. The ACR guideline [14] had more disease specific general principles and included statements about the need for payers not to influence some treatment decisions. The English (Royal College of Physicians) [29] guideline was most specific about its audience, but it was designed to be part of the government-funded National Health Service. Other guidelines, such as the APLAR guideline [15] highlighted the diversity of patients managed in the areas they represent and the potential differences from Western countries.

### Intended audiences

Twenty guidelines outlined, to a greater or lesser extent, their intended audience [14–17, 19–31, 33–35]. All 20 indicated they were mainly aimed at clinicians; the Australian (Royal Australian College of General Practitioners) indicated their guidelines [16] were specifically intended for GPs. Other guidelines included broader ranges of medical specialists and other health care professionals involved in the management of RA. Guidelines were sometimes intended to provide information for a broader range of readers: 6 guidelines [19–21, 23, 29, 34] included a range of administrative staff including commissioners and payers of healthcare; 7 guidelines [14, 19–21, 23, 29, 34] included patients and in some cases patient groups. An example of a guideline with a broad audience is English (Royal College of Physicians) guidance [29] which spanned all healthcare professionals, people with RA and their carers, patient support groups, commissioning organisations and service providers.

### Structure

13/22 guidelines dealt with specific questions or recommendations [14, 16, 17, 19, 21–25, 28, 29, 34, 35]; the average number was 20 (range 10–37). Some of these guidelines had specific structures which were replicated across questions; for example the Canadian guideline [21] for each question included the recommendation, the supporting evidence and the barriers to implementation. The other 9/22 guidelines focused on different themes or areas [15, 18, 20, 26, 27, 30–33] which incorporated a number of related issues; the average number was 6 (range 3–12). Some of these guidelines also had specific structures replicated across themes; for example the English (Royal College of Physicians) guideline [29] had summaries of the evidence, sections from evidence to recommendations and then one or more recommendations for each of the themes it considered.

### Assessments

18/22 guidelines [14, 15, 17, 21–35] recommend regular assessments using a variety of clinical assessments based on the Outcome Measures in Rheumatology (OMERACT) core dataset [39] using composite indices. These all recommended using the disease activity score for 28 joints (DAS28) [40]. In addition 14 aslo recommended simple disease activity index (SDAI) and 13 recommended Clinical Disease Activity Index (CDAI) [41]. Two guidelines recommended other assessments – the Patient Activity Scale (PAS) [42] and Routine Assessment Of Patient Data Index (RAPID3) [43]. None of the guidelines specifically recommended one composite index over another. The importance of assessing disability was considered by most guidelines. The recommendations varied more widely on how to do this and 10/22 guidelines recommended regularly assessing disability [15, 17, 21, 25–27, 29, 31–33]: 9 of these recommended using the Health Assessment Questionnaire (HAQ) [44]; the Canadian guidelines did not specifically suggest assessing HAQ regularly [21].

The importance of frequent assessment is stressed in most guidance. Some guidelines gave relatively specific suggestions. For example EULAR guidelines recommend assessing patients every 1 to 3 months, at least in the early stages of their RA. Many guidelines indicated patients should be assessed by rheumatologists at least annually. The English (Royal College of Physicians) guideline gives a very specific recommendation for annual review. The ACR guideline recommended annual assessments of function.

### Remission and other targets

Twenty guidelines recommended remission as a treatment target and 16 guidelines recommended using low disease activity as an alternative target (Table 2). Two guidelines recommend aiming to suppress inflammation: the British Columbia guideline [18] concluded that the objective of treatment is to “suppress all inflammation”, implying this is joint inflammation; the British Society For Rheumatology established RA guideline [19] recommended “suppressing inflammation” indicating this was to limit disease progression.

**Table 2:** Recommended treatment targets

Guideline	Treatment Target			Remission Definitions				Treat Moderate Disease
	Remission	LDA	Suppress Inflammation					
1. American [14]	Yes	Yes	–	SDAI	Boolean			Yes
2. APLAR [15]	Yes	Yes	–	DAS28	SDAI	CDAI	Boolean	Yes
3. Australian [16]	Yes	–	–	–	–	–	–	Yes
4. Brazilian [17]	Yes	Yes		DAS28	SDAI	CDAI	–	Yes
5. British Columbia [18]	–	–	Yes	–	–	–	–	–
6. British Society For Rheumatology: Established [19]	Yes	–	Yes	–	–	–	–	–
7. British Society For Rheumatology: Early [20]	–	–	–	–	–	–	–	–
8. Canadian [21]	Yes	Yes	–	DAS28	SDAI	CDAI	Boolean	Yes
9. EULAR [22]	Yes	Yes	–	SDAI	Boolean	–	–	Yes^a^
10. French [23]	Yes	Yes	–	DAS28	SDAI	CDAI	Boolean	Yes
11. German [24]	Yes	Yes	–	DAS28	–	–	–	Yes^a^
12. Hong Kong [25]	Yes	–	–	DAS28	–	–	–	Yes^a^
13. Indian [26]	Yes	–	–	–	–	–	–	Yes
14. Latin American [27]	Yes	Yes	–	DAS28	–	–	–	–
15. Mexican [28]	Yes	Yes	–	DAS28	–	–	–	Yes
16. England [29]	Yes	Yes	–	DAS28	–	–	–	–
17. Scotland [30]	Yes	Yes	–	DAS28	–	–	–	Yes
18. South African [31]	Yes	Yes	–	SDAI	–	–	–	Yes
19. Spanish [32]	Yes	Yes	–	DAS28	CDAI	Boolean	–	Yes^a^
20. Swedish [33]	Yes	Yes	–	DAS28	SDAI	CDAI	–	Yes
21. Treat to Target [34]	Yes	Yes	–	DAS28	SDAI	CDAI	Boolean	Yes
22. Turkish [35]	Yes	Yes	–	–	–	–	–	Yes

*LDA* Low disease activity

^a^Treatment of moderate RA is implied rather than definitively stated

Remission was defined in various ways, in keeping with current international criteria [45]. DAS28-defined remission was recommended in 13 guidelines, SDAI in 9, CDAI in 7 and Boolean in 6. There were 6 guidelines which did not give any criteria for assessing the presence of remission. In addition many guidelines emphasised the importance of minimising disability, minimising progressive joint damage and maximising quality of life, though these were less explicit management goals.

All guidelines recommend treating active RA. There was less unanimity about treating moderately active disease. Thirteen guidelines made specific recommendations about treating moderate disease. Four guidelines gave implied guidance about treating moderate disease in that they indicated what treatment policies were needed until patients achieved remission. Five guidelines made no recommendations about treating moderate disease.

### Prognostic assessments to guide treatment decisions

Sixteen guidelines specifically included assessments of prognostic factors to help guide management decisions about treatments [15–18, 21–28, 31–33, 35]. All these 16 guidelines recommended using anti-citrullinated protein antibodies (ACPA); 14 guidelines recommended using rheumatoid factor (RF) [15–17, 21–28, 31–33]; 15 guidelines recommended using x-ray erosion [15, 16, 21–27, 31–33, 35]; and 9 guidelines recommended using high disability or extra-articular disease [21, 25–28, 31–33, 35]. These recommendations are summarised in Table 3. The guidelines including prognostic assessments all recommended considering more intensive treatment with conventional DMARDs and biologic DMARDs in those patients with poor prognostic features. They gave variable details of exactly how this should be achieved.

**Table 3:** Recommended composite disease activity assessments and prognostic assessment to guide treatment

Guideline	Composite Disease Activity Assessments					Prognostic Assessments				
	PAS	RAPID3	CDAI	SDAI	DAS28	RF	ACPA	X-ray Erosions	Poor Function	Extra-Articular Disease
1. American [14]	Yes	Yes	Yes	Yes	Yes					
2. APLAR [15]	–	–	Yes	Yes	Yes	Yes	Yes	Yes		
3. Australian [16]	–	–	–	–	–	Yes	Yes	Yes		
4. Brazilian [17]	–	–	Yes	Yes	Yes	Yes	Yes	Yes		
5. British Columbia [18]	–	–	–	–	–		Yes	Yes		
6. British Society For Rheumatology: Established [19]	–	–	–	–	–					
7. British Society For Rheumatology: Early [20]	–	–	–	–	–					
8. Canadian [21]	Yes	Yes	Yes	Yes	Yes	Yes	Yes	Yes	Yes	Yes
9. EULAR [22]			Yes	Yes	Yes	Yes	Yes	Yes		
10. French [23]			Yes	Yes	Yes	Yes	Yes	Yes		
11. German [24]	–	–	–	–	Yes	Yes	Yes	Yes		
12. Hong Kong [25]	–	–	Yes	Yes	Yes	Yes	Yes	Yes	Yes	Yes
13. Indian [26]	–	–	Yes	Yes	Yes	Yes	Yes	Yes		Yes
14. Latin American [27]	–	–	–	–	Yes	Yes	Yes	Yes	Yes	Yes
15. Mexican [28]			Yes	Yes	Yes	Yes	Yes			Yes
16. England [29]	–	–	–	–	Yes					
17. Scotland [30]	–	–	Yes	Yes	Yes					
18. South African [31]	–	–	–	Yes	Yes	Yes	Yes	Yes	Yes	Yes
19. Spanish [32]	–	–	Yes	Yes	Yes	Yes	Yes	Yes	Yes	Yes
20. Swedish [33]	–	–	Yes	Yes	Yes	Yes	Yes	Yes	Yes	Yes
21. Treat to Target [34]	–	–	Yes	Yes	Yes					
22. Turkish [35]	–	–	–	–	Yes		Yes	Yes	Yes	

*RF* Rheumatoid factor, *ACPA* Anti-citrullinated protein antibody

### Initial conventional DMARD recommendations

Twenty one guidelines dealt with the management of early RA; all of these recommended starting conventional DMARDs as soon as possible after diagnosis. Methotrexate, which is often described as the “anchor” drug for RA, was recommended for most patients in 19/22 guidelines [14–17, 20–29, 31–35] (Table 4). In 13/22 guidelines there was consideration of the relative benefits and risks of oral and subcutaneous methotrexate [14, 17, 20–24, 27, 29, 31–33, 35]; however, the approach taken to this issue varied considerably and there was no obvious consensus across guidelines about when best to use parenteral methotrexate.

**Table 4:** Drug treatment recommendations

Guideline	DMARDs					Biologics			Symptomatic Treatments	
	MTX	Others	Combinations	JAK Inhibitors	Glucocorticoids (steroids)	First	Subsequent	Tapering	NSAIDs	Pain
1. American [14]	Yes	Yes	Yes	Yes	Yes	Yes	Yes	Yes	–	–
2. APLAR [15]	Yes	Yes	Yes	Yes	Yes	Yes	Yes	Yes	Yes	–
3. Australian [16]	Yes	Yes	Yes	–	Yes	Only for specialists			Yes	Yes
4. Brazilian [17]	Yes	Yes	Yes	–	Yes	Yes	Yes	Yes	Yes	–
5. British Columbia [18]	Yes	Yes	Yes	–	Yes	Only for specialists			Yes	Yes
6. British Society For Rheumatology: Established [19]	Generic DMARDs		–	–	–	Generic biologics			Yes	Yes
7. British Society For Rheumatology: Early [20]	Generic DMARDs		Yes	–	Yes	Generic biologics		Implied	Yes	Yes
8. Canadian [21]	Yes	Yes	Yes	–	Yes	Yes	Yes	Yes	–	–
9. EULAR [22]	Yes	Yes	–	Yes	Yes	Yes	Yes	Yes	–	–
10. French [23]	Yes	Yes	Yes	–	Yes	Yes	Yes	Yes	–	–
11. German [24]	Yes	Yes	Yes	–	Yes	Yes	Yes	–	–	–
12. Hong Kong [25]	Yes	Yes	Yes	–	Yes	Yes	Yes	–	–	–
13. Indian [26]	Yes	Yes	Yes	–	Yes	Yes	Yes	–	Yes	Yes
14. Latin American [27]	Yes	Yes	Yes	–	Yes	Yes	Yes	–	Yes	Yes
15. Mexican [28]	Yes	Yes	Yes	Yes	Yes	Yes	Yes	–	Yes	Yes
16. England [29]	Yes	Yes	Yes	–	Yes	Yes	Yes	–	Yes	Yes
17. Scotland [30]	Yes	Yes	Yes	–	Yes	Yes	Yes	–	Yes	Yes
18. South African [31]	Yes	Yes	Yes	–	Yes	Yes	Yes	–	Yes	Yes
19. Spanish [32]	Yes	Yes	Yes	–	Yes	Yes	Yes	–	Yes	Yes
20. Swedish [33]	Yes	Yes	Yes	–	Yes	Yes	Yes	Yes	–	–
21. Treat to Target [34]	Generic DMARD treatments			–	Yes	Generic biologics		Implied	–	–
22. Turkish [35]	Yes	Yes	Yes	–	Yes	Yes	Yes	Yes	–	–

When there are contraindications to methotrexate or if there are clinically significant adverse events to methotrexate all 19 guidelines that suggested methotrexate as initial treatment recommend considering alternative conventional DMARDs. Sulfasalzine, leflunomide and hydroxychloroquine were all considered potentially appropriate; there was no consistent pattern in these recommendations. Other rarely used conventional DMARDs, such as azathioprine, though not excluded were not specifically recommended.

Three guidelines considered DMARDs generically without giving recommendations about which drugs to use; these were the British Guidelines for established [19] and early RA [20] and the EULAR treat to target guidance [34]. These three guidelines focussed on the overall strategy for managing RA rather than the best individual treatment options and so consequently did not provide recommendations about specific drugs.

The way individual guidelines outlined the initial treatment for RA varied considerably. EULAR guidelines recommend that methotrexate should be part of the first treatment strategy. ACR guidelines recommend that DMARD monotherapy is generally more acceptable and better tolerated than combination DMARD therapy and that methotrexate should be the preferred initial DMARD for most early RA patients. Canadian guidelines recommend that initial combination therapy with traditional DMARD should be considered, particularly in patients with poor prognostic features, moderate-high disease activity and in patients with recent-onset disease. English (Royal College of Physicians) guidelines recommended that in people whose RA is active, patients should be offered a combination of DMARDs (including methotrexate, at least one other DMARD, plus short term glucocorticoids) as first-line treatment.

### Combinations of conventional DMARDs

Twenty guidelines considered the use of combinations of conventional DMARDs; 19 of these guidelines recommended using them in some patients [14–18, 20, 21, 23–33, 35]. They were recommended when patients failed to respond fully to DMARD monotherapy and that biologics were not necessarily indicated. Specific Combinations of conventional DMARDS were recommended by 12/22 guidelines [14, 15, 17, 21, 23–28, 31, 33]: these combinations comprised methotrexate with sulfasalazine and hydroxychloroquine or methotrexate with leflunomide in 9 guidelines; 2 guidelines omitted leflunomide from combinations [23, 33] and one guideline recommended chloroquine instead of hydroxychloroquine [31]. One guideline, from England, recommended initial combinations of conventional DMARDs [29], though it did not specify which drugs to use.

The one exception was the EULAR guidelines which do not specifically recommend using them. However, EULAR did not exclude their use, and mention them briefly. The EULAR guidelines also provide an extensive commentary on the divergence of expert opinion on this issue, highlighting potential toxicities and difficulties dissociating the impact of methotrexate, short-term glucocorticoids (steroids) and other conventional DMARDs in combinations.

### Janus Kinase inhibitors

Only 4 guidelines consider the use of Janus Kinase inhibitors; this mainly reflects whether they were developed after these drugs became available. Those guidelines that consider them recommend their use as an alternative to biologics in some patients with established RA. They are usually recommended to be used in combination with methotrexate.

### Glucocorticoids (steroids)

Twenty guidelines recommended using glucocorticoids in some RA patients; these were usually patients with early RA who were starting DMARD treatment. In the main only short-term courses of low dose glucocorticoids (steroids) were recommended. The EULAR treat to target guideline implied glucocorticoids (steroids) should be used within the treatment strategy in some patients but did give any recommendations about specific therapies. The British guidelines for established RA did not consider glucocorticoids (steroids). In addition some guidelines gave advice about the role of glucocorticoids (steroids) in specific clinical settings, particularly in the management of some comorbidities.

### Biologic DMARD

Twenty guidelines made recommendations about using biologics. Three guidelines made generic recommendations about biologics and the other 17 that dealt with them considered individual biologics and classes of biologics. The 2 guidelines that did not were for primary care clinicians who should not usually prescribe these treatments. All the guidelines that dealt with biologics recommended their use in patients who had failed to respond to conventional DMARDs, particularly methotrexate. They also recommended using them in combination with methotrexate whenever possible. Most guidelines did not make specific recommendations about using one class of biologics preferentially. However, some guidelines such as the Canadian ones, recommend using tumour necrosis factor inhibitors as an initial biological treatment. In patients who have continuing disease activity despite biologic treatment or adverse events to biologics starting an alternative biologic was recommended. In most instances no particular sequences of biologics were recommended in the different guidelines.

### Biologic DMARD tapering

Eight guidelines recommended considering tapering biologic treatment in patients who had achieved sustained good responses and remissions. A further two guidelines implied this was appropriate without giving detailed recommendations.

### Symptomatic treatment

Thirteen guidelines made recommendations about the use of NSAIDs and 12 about using analgesics to control symptoms. Those guidelines which consider the use of NSAIDs invariably focus on minimising exposure to these treatments. For example the Scottish Guidelines suggest using the lowest NSAID dose compatible with symptom relief, and indicate that treatment should be reduced and if possible withdrawn as soon as possible and that gastro-protection should be included when using them. When analgesics such as paracetamol were mentioned for symptom relief though the evidence supporting their use is noted to be minimal by current standards.

## Discussion

Our overview of 22 different RA management guidelines shows that several general principles transcend the majority of them. Firstly DMARDs should be started as soon as possible after the diagnosis has been established. Secondly disease activity should be regularly monitored using composite indices such as DAS28, which relates to our initial aim which was our initial specific question. Thirdly methotrexate is the best initial treatment, and that this can be usefully supplemented with short-term glucocorticoid (steroid) therapy. Fourthly biologic DMARDs should be given to patients with persistently active disease who have already received methotrexate and, in some instances another conventional DMARD. These principles relate to another of our specific questions. Fifthly remission or low disease activity is a suitable target and that treatment can be tapered in patients who have achieved sustained remissions. This principle relates to our final specific question. We consider that applying these general principles to RA management in all clinical settings is likely to achieve good overall clinical outcomes.

There is considerable uncertainty about the value and place for using combinations of conventional DMARDs. The most recent EULAR guidance is particularly uncertain about its value. Other guidance including the ACR guidance is more definite it is perspective. The reasons for this difference are unclear. In part it may be presentational; EULAR guidance does not exclude using such combinations and ACR guidance does not explicitly recommend them; consequently much of the apparent difference may represent the way in which the information is presented. There has been correspondence about this particular aspect of the EULAR guidelines [46, 47]. However, the balance of opinion in these various guidelines favours the use of combinations of conventional DMARDs in some patients. Interestingly, recent guidance from NICE in a multiple technology appraisal (a type of assessment we excluded from this systematic review) recommended only starting biologics in patients with disease that had not responded to intensive therapy with a combination of conventional DMARDs [48]. These perspectives were from expert groups who had considered the same evidence in detail and they show the divergence of expert views when assessing clinical research findings.

There is also relatively little overall consensus about treating moderately active RA. The ACR guidance makes the strongest recommendation on this point. Other guidance has either not considered it or may have been published prior to much evidence becoming available. Despite the limitations of explicit recommendations, those guidelines which consider moderate disease recommend treating it intensively.

The guidelines differ in the formality of their approach and in the extent of systematic reviews commissioned specifically for them. The EULAR, ACR and Royal College of Physicians guidelines were the most detailed and involved the greatest amount of preparatory work including a number of detailed systematic reviews. Specialist rheumatologists were involved in almost all guidelines; varying numbers of other experts and patients were involved. The impact that these non-rheumatologists would be able to make to the guidelines was uncertain.

The limitations of clinical guidelines have been described in detail [49–52]. We do not intend to consider the relative strengths and weakness of guidelines in general. However, one particular challenge with the current published guidelines is that only 8/22 specifically followed a nationally or internationally agreed approach to ensure they were of high quality. Future guidelines ought to explicitly adopt one of these quality methods. In RA the overall the degree of agreement between the guidelines is striking and exceeds the differences between them. As health care is not universally uniform it is inevitable national groups would wish to have their own local guidelines, which reflect the arrangements of their medical systems. The overall impact of the guidelines is difficult to establish. As the various updates of ACR and EULAR guidelines have high citation rates on bibliometric systems it seems likely they are used by many groups. Some guidelines have immediate practical implications. For example technology appraisals by NICE, though outside our remit, have been crucial for ensuring patients have access to high cost therapies. It is likely guidelines achieve this goal more globally, and the appearance of many guidelines reflects the major changes in drug therapy for RA in recent years.

Our own assessment of RA guidelines has its own limitations. Firstly, some of the guidelines were developed over 10 years or longer and the older ones cannot have included the more recent clinical evidence. Therefore comparisons need to take this into account. Secondly, there are different types of guidelines. We have included general ones. Many others focus on single drugs or treatment modalities including surgery. It is difficult to draw a clear line between which ones to include and which to omit. Not all experts would necessarily agree with our approach to inclusion. Thirdly, we have only provided a narrative assessment of them. They are too diverse in their approaches to allow any synthesis of their various conclusions and recommendations. Fourthly we have focussed on issues in the guidelines we consider to be of most importance. Other experts may have considered different aspects of the guidelines in more detail and overlooked some of the matters we have dealt with. Finally, systematic reviews of guidelines are not one of the current PRISMA extensions [53] though we anticipate they will be included in subsequent updates. Consequently we did not register our protocol; however, several other recent systematic reviews have evaluated different guidelines using similar approaches to our own, such as the report by Jollife et al. on stroke rehabilitation guidelines [13] Systematic reviews of guidelines differ from both scoping [54] and umbrella reviews [55].

Our analysis shows several things. Firstly, the recommendations in the guidelines are broadly similar, though they differ in some points of detail; for example the use of combinations of conventional DMARDs. Such minor variations most likely reflect the challenges in balancing evidence of benefits against evidence of risks. Secondly, although guidelines deal with the same issue, they bring together different groups of experts and it is likely the production of guidelines enhances clinical practice. Consequently multiple guidelines appear to be needed. Thirdly, although it is difficult to judge accurately the impact of guidelines on clinical practice, there is evidence that RA outcome have improved significantly during the last 10–20 years and in part this is likely to reflect the impact of guidelines in improving the quality of clinical practice. Finally, as new treatments are introduced, particularly new JAK inhibitors, guidelines will need to be continually updated and, potentially produced by different groups.

We anticipate that many of the existing guidelines will be updated in future years. We believe it important to do so to maintain their relevance to clinical practice. The frequency of review will reflect the timing of new clinical information. Looking back at the earliest guidelines from the 1990s [1–3] shows just how much clinical practice has changed over the years, indicating the need for guidance to be updated. We consider there are two ways in which the process of developing guidelines could be improved. Firstly, there guideline development should conform with one of the published quality standards; whilst there is no reason to prefer one standard over another, it seems worthwhile to adopt one of them. Secondly, guidelines should incorporate divergent views, when there is no universally agreed answer. The controversy about the value of combinations of conventional DMARDs highlights this issue.

One important role of guidelines is to suggest potential future research questions. Our own research in the TITRATE research programme, of which this systematic review in a single component, was based on the absence of evidence on the benefits of intensive management in moderately active RA [56]. Interestingly, though the clinical research evidence has changed little on this aspect of treat to target, current guidelines often recommend treating moderately active RA intensively, showing the way in which guidelines interpret the evidence in very different ways.

## Conclusions

Although a number of differences exist between guidelines, there are some general principles. These include starting DMARDs soon after diagnosis; methotrexate should be used first line; disease activity should be monitored regularly; biologics therapies should be used where there is persistently active disease; and remission or low disease activity is the preferred target.

## Data Availability

All data generated or analysed during this study are included in this published article.
